# Assessment of Zinc-Bound Phosphate-Based Glass-Coated Denture-Relining Material with Antifungal Efficacy for Inhibiting Denture Stomatitis

**DOI:** 10.3390/nano12173048

**Published:** 2022-09-02

**Authors:** Sang-Hwan Oh, Yun-Sook Jung, Myung-Jin Lee

**Affiliations:** 1 Department of Dental Hygiene, Konyang University, Daejeon 35365, Korea; 2 Department of Dental Hygiene, College of Science & Technology, Kyungpook National University, Sangju 37224, Korea; 3 Department of Dental Hygiene, Division of Health Science, Baekseok University, Cheonan 31065, Korea

**Keywords:** antifungal efficacy, biocompatibility, *Candida albicans*, dental materials, surface characterization, zinc-bound phosphate-based glass

## Abstract

This study investigated the surface properties, biocompatibility, and antifungal activity against *Candida albicans* of a denture-relining material coated with zinc-bound phosphate-based glass. First, zinc-bound phosphate-based glass was fabricated. A polymerized denture-relining disk was coated with zinc-bound phosphate-based glass (2%, 4%, and 6%). The surface properties of the control and experimental groups were measured, including the wettability, microhardness, color difference, and gloss. The biocompatibility was evaluated using the MTT assay according to ISO 10993-5. The antifungal activity was investigated by counting the number of colony-forming units of *Candida albicans*. The results were analyzed using a one-way ANOVA and Tukey’s test (*p* = 0.05). The results of this study indicate that, despite the antimicrobial effect of zinc-bound phosphate-based glass, a coated denture-relining material does not degrade the surface properties and biocompatibility. Therefore, this novel material is considered promising for use as a dental material with antimicrobial properties that can potentially prevent denture stomatitis.

## 1. Introduction

Improvements in medical technology and living standards as well as a greater focus on health have led to a steady increase in human lifespan [1]. Consequently, the global population is aging [2], driven by improvements in quality of life. The rapid growth in the mean population age has seen the elderly population growing faster than the young population [3]. For this reason, it is necessary to maintain physical health, and one such measure is oral health management [2].

Many older adults use dentures [1,4]. Acrylic resin, a material for making dentures, has been used in dentistry for a long time and is a dental material that has been improved in terms of its physical properties and biocompatibility [5]. However, denture-relining materials have relatively poor antimicrobial properties, which can cause denture stomatitis, a common oral mucosal disease [6]. Denture stomatitis is associated with various factors, including improper denture mounting, bacterial mucosal infection, and denture plaque deposition [7,8]. Fungal adhesion and colonization can easily be induced on the surface of the denture base, which can cause denture stomatitis, a chronic inflammatory condition [9,10]. Denture stomatitis is associated with high morbidity, especially in denture wearers over 65 years of age, and the most common causative agent is *Candida albicans* [11]. It is known that fungi with hydrophobized cell walls can attach to a polymer surface, such as a denture base [12]. The treatment of *Candida* includes the disinfection of dentures, the application of antifungal agents, and medication [13]. However, high-dose drug administration is required, and elderly patients exhibit a high recurrence rate [14]. In addition, the ability of the denture-using elderly to maintain good oral hygiene is low, and it is difficult to expect correct denture management; preventing this disease is therefore vitally important [15]. According to previous reports, the use of chemical disinfectants to sterilize dentures led to changes in color, increased surface roughness, and adverse effects on the skin and eyes [16,17]. In addition, there have been reports that the use of antimicrobial treatments causes health problems associated with increased antimicrobial resistance [18].

Recently, research on natural inorganic antimicrobial substances has been conducted to overcome the resistance caused by antimicrobial abuse [19,20], including zinc, which is toxic to oral bacteria [21,22]. Zinc is gaining popularity as a safe inorganic antibacterial substance that is tissue-friendly with low cytotoxicity and is unlikely to contribute to antimicrobial resistance [21]. The inhibition of plaque formation and metabolism by zinc has been well-documented [23]. Previous studies have suggested that zinc exhibits antibacterial effects by inhibiting acid production and adsorbing onto bacterial cell walls [24,25]. However, although zinc may exhibit antibacterial activity, conventional delivery methods provide only short-term effects, which appear to limit the effectiveness of zinc. Therefore, this discrepancy in the role of zinc has induced the use of zinc-doped phosphate-based glasses (Zn-PBGs) as independent controlled delivery agents, which have been reported to remain stable and effective for long-term use. Bioglass-based materials, including phosphate-based or phosphate-containing glasses, have also been used in this context [26]. The primary advantage of these materials is their negligible cytotoxicity. Phosphate-based glass (PBG) exhibits composition-dependent chemical durability, which limits its application in aqueous solutions [27]. Recently, the mechanism of its cytotoxicity was determined to be from the release of ions due to its degradation in solution. It has been revealed that the chemical durability of Zn-PBG is dependent on its composition [25,28]. ZnO is non-toxic, and there are reports of its antibacterial activity through the generation of reactive oxygen species on the oxide surface, which disrupts the integrity of the bacterial membrane. Additionally, Jones et al., compared the antibacterial activities of selected inorganic materials, among which ZnO exhibited significant growth inhibition [29]. Therefore, the combination of PBG with ZnO is promising.

Although studies on the antimicrobial properties of zinc-doped composites have been conducted in several academic fields [21,25], studies on denture-relining materials using zinc-doped phosphate glass have not yet been reported. In this study, zinc-doped phosphate glass was developed and introduced into denture-relining materials. The antimicrobial properties of flowable composite resins with added Zn-PBG were investigated for their suitability for dentistry. This study also investigated the mechanical and ion-releasing properties and antimicrobial activities of Zn-PBG-containing bioactive flowable resin composites. The null hypotheses of this study were as follows: (1) a denture-relining material containing zinc-doped phosphate glass would not result in significant differences in the surface properties compared to the control, (2) a denture-relining material containing zinc-doped phosphate glass would not result in significant differences in cell cytotoxicity compared to the control, and (3) a denture-relining material containing zinc-doped phosphate glass would not result in significant differences in antifungal activity compared to the control.

## 2. Materials and Methods

### 2.1. Preparation of Zinc-Doped Phosphate Glass

Batches of zinc-doped phosphate glass powder were fabricated by mixing specific weights of high-purity P_2_O_5_ (42 mol%), CaO (25.2 mol%), Na_2_O (16.8 mol%), and ZnO (16 mol%) powders in a tubular shaker-mixer. The blended batch was melted in an alumina crucible at 1100 °C for 1 h in an electric furnace. The melted glass was then quenched at room temperature to obtain a glass cullet, ground in an alumina mortar, and pulverized under dry conditions using a planetary mono mill (Pulverisette-7; Fritsch, Idar-Oberstein, Germany).

### 2.2. Fabrication of Control and Experimental Specimens

Zn-PBG powder was incorporated in a light-curable coating resin material (Plaquit Solution, Dreve, Unna, Germany) and mixed using a magnetic stirrer for 48 h. The following control and experimental groups were prepared: 0 wt% (control), 2 wt% (Zn-PBG-2%), 4 wt% (Zn-PBG-4%), and 6 wt% (Zn-PBG-6%). A Teflon mold (10 mm × 10 mm × 0.1 mm) was positioned on a glass slide covered with transparent polyethylene. The powder and liquid components of the autopolymerizing denture-relining material (Tokuyama Rebase II, Tokuyama, Japan) were mixed according to the manufacturer’s instructions. The powder and liquid were mixed until reaching a doughy consistency. The Teflon mold was filled with denture-relining material and covered with a polyester film and a glass slide. After polymerization, the denture-relining material was separated from the Teflon mold. Finally, 10 µL of the experimental coating resin was spread on the prepared denture-relining material with a microbrush.

### 2.3. Surface Wettability

A contact angle analyzer (Phoenix 300, SEO, Suwon, Korea) was used in this study to measure the surface wettability of each specimen. Five microliters of distilled water were dropped on the samples randomly, and the contact angles of these specimens were measured after 10 s of contact.

### 2.4. Surface Microhardness

The Vickers hardness was investigated using a microhardness tester (DMH-2, Matsuzawa Seiki Co., Ltd., Tokyo, Japan) under an established load of 0.09 MPa for 20 s using a pyramidal-shaped diamond indenter [4]. Measurements were performed at five points on each specimen, and the average values were calculated.

### 2.5. Color Characteristics

The color differences were evaluated using a spectrophotometer (CM-3500d; Minolta, Kyoto, Japan). A white plate was used for standard calibration before measuring each specimen. The specimens were assessed according to the obtained a*, b*, and L* values, and ΔE* values were calculated according to the CIE Lab system. The color measurements were averaged at various locations on each specimen.

### 2.6. Gloss

Gloss specimens were prepared using a Teflon mold with a 2 mm height and a 10 mm diameter. Two disk-shaped specimens were prepared for each, and eight specimens were used in this study. After fabrication, the specimens were polished sequentially using 800–2000-grit silicon carbide paper. Gloss was analyzed using a Novo-Curve glossmeter (Novo-Curve, Rhopoint Instrumentation, East Sussex, UK). After calibrating the glossmeter, five points on the flat surface of each specimen were randomly measured at an angle of 60°.

### 2.7. Scanning Electron Microscopy Surface Images

The samples were sputter-coated with platinum to facilitate the observation of the sample surface. Scanning electron microscopy (SEM, Merin, Carl Zeiss, Oberkochen, Germany) images were obtained at an accelerating voltage of 15 kV and a magnification of 50×.

### 2.8. Cell Viability Test

An MTT (3-(4,5-dimethylthiazol-2-yl)-2,5-diphenyl tetrazolium bromide) assay was performed using L929 murine fibroblasts (Korean Cell Line Bank, Seoul, Korea) to evaluate the biocompatibility of the control and experimental groups, according to the guidelines established in *Biological evaluation of medical devices—Part 5: Tests for* in vitro *cytotoxicity,* ISO 10993-5:2009. Suspensions of L929 cells were adjusted to a density of 1 × 10^4^ cells/mL, and 100 µL of the cell suspension was incubated with a denture base resin specimen for 24 h [4]. Then, 100 µL of the solution was replaced with 50 µL of 1% MTT solution. The plate was stored in an incubator at 37 °C for 3 h. The MTT solution was removed, and 100 µL of isopropanol was added to solubilize the formazan product. The absorbance was measured at 570 nm using an ELSA microplate spectrophotometer (Epoch, BioTek, Winooski, VT, USA).

### 2.9. Antifungal Test

Antifungal tests were carried out using *Candida albicans* (ATCC 10231) cultured in a yeast and mold medium (YM, Becton Dickinson and Co., Franklin Lakes, NJ, USA) in an incubator at 37 °C for 24 h. The test specimens were sterilized using ethylene oxide. The fungal cultures were diluted to obtain an optical density (OD_600_) of 0.4–0.6, and the absorbance was measured at 600 nm using an optical density reader (Epoch, BioTek, Winooski, VT, USA). Then, 1 mL of the fungal culture suspension (1 × 10^8^ cells/mL) was placed on each specimen at 37 °C. After 24 h, the samples were washed with distilled water, and the attached fungi were resuspended in 1 mL of YM by sonication (SH-2100; Saehan Ultrasonic, Seoul, Korea) for 5 min. Next, 100 µL of the *C. albicans* suspension was uniformly spread on YM agar plates, and the number of colony-forming units (CFU) was evaluated.

### 2.10. Statistical Analysis

Five independent replicates were performed for all experiments, and data were calculated as averages and standard deviations using statistical software (IBM SPSS, version 25.0, IBM Korea Inc., Armonk, NY, USA). The results were analyzed using a one-way analysis of variance followed by Tukey’s statistical test. The significance level was set at *p* < 0.05.

## 3. Results and Discussions

According to previous studies, the quality of life related to oral health in the elderly improved after denture prosthesis use, and denture satisfaction was directly related to the quality of life related to oral health [2]. Denture prosthesis materials are used in the oral cavity, where plaques and various bacteria are present [12]. Therefore, there have been many studies on the adhesion of bacteria and plaque to polymer restorative materials, including denture bases [8,14,20]. Plaque and bacterial adhesion affect the discoloration of restorations, denture stomatitis, and general health [3,18]. Inhibiting the microbiome prevents denture stomatitis; however, the denture base does not have antifungal properties [1]. Ideally, dentures should exhibit stable color and surface properties without irritation of the oral mucosa [4,9]. Therefore, we attempted to develop a denture base material with established antibacterial effects and investigate its surface properties, cytotoxicity, and antifungal effects. To the best of our knowledge, this is the first study to develop a denture-relining material coated with zinc-doped phosphate glass as an antifungal agent.

Our previous study reported that the X-ray diffractometry diffraction pattern of Zn-PBG was non-crystalline in nature, consistent with that of a typical glass, and that the distribution was bimodal, as identified with a particle size analysis [25]. The control of surface properties is necessary for clinical application. Therefore, in this study, the surface wettability, microhardness, color difference, and gloss were analyzed and compared with those of the control group.

**Figure 1:**
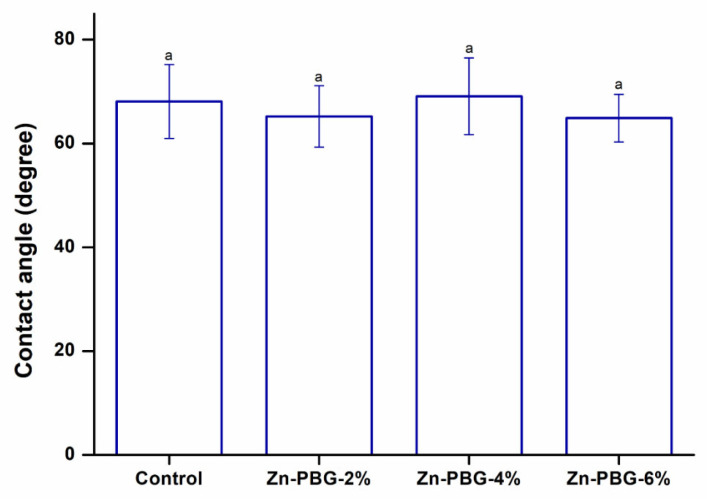
Contact angle of the control, Zn-PBG-2%, Zn-PBG-4%, and Zn-PBG-6% groups. The lowercase letter indicates no significant difference between the control and experimental groups (*p* > 0.05).

The results of the surface wettability tests are shown in Figure 1. The contact angles among the groups showed no significant differences (*p* > 0.05). It has been reported that when the surface becomes more hydrophobic, the adhesion of bacteria improves [30]. The material used in this study did not change the surface hydrophobicity and showed the same result as the control; therefore, the contact angle of the surface was not affected. It is also clinically important that wettability does not change because dentures are always used in the wet environment of the oral cavity [31].

Surface hardness is a key factor that affects the wear resistance of prostheses [4]. When surface hardness is low, the surface can be easily deformed. It has been shown that the denture surface is affected when cleaning dentures; mechanical cleaning techniques and denture cleansing agents adversely affect the surface by increasing wear and decreasing the stability of dental prostheses [1].

**Figure 2:**
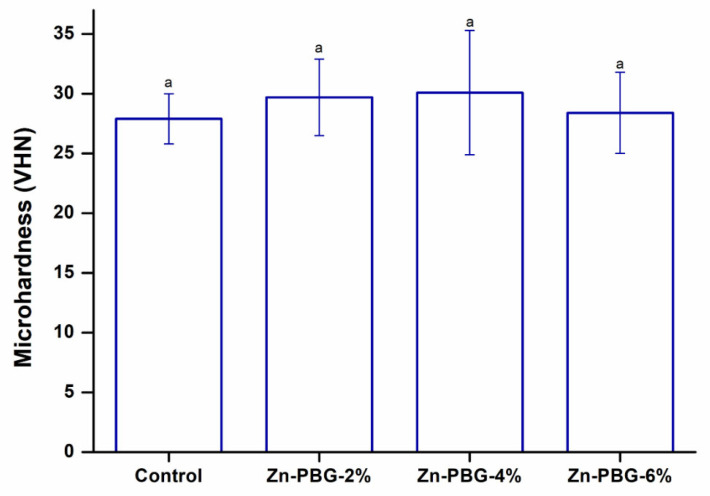
Microhardness of the control, Zn-PBG-2%, Zn-PBG-4%, and Zn-PBG-6% groups. The lowercase letter indicates no significant difference between the control and experimental groups (*p* > 0.05).

The results of the microhardness tests are shown in Figure 2. The microhardness values among the groups were not significantly different (*p* > 0.05). The hardness values of the control, 2, 4, and 6 wt% Zn-PBG groups were 27.9 ± 2.1, 29.7 ± 3.2, 30.1 ± 5.2, and 28.4 ± 3.4, respectively.

**Figure 3:**
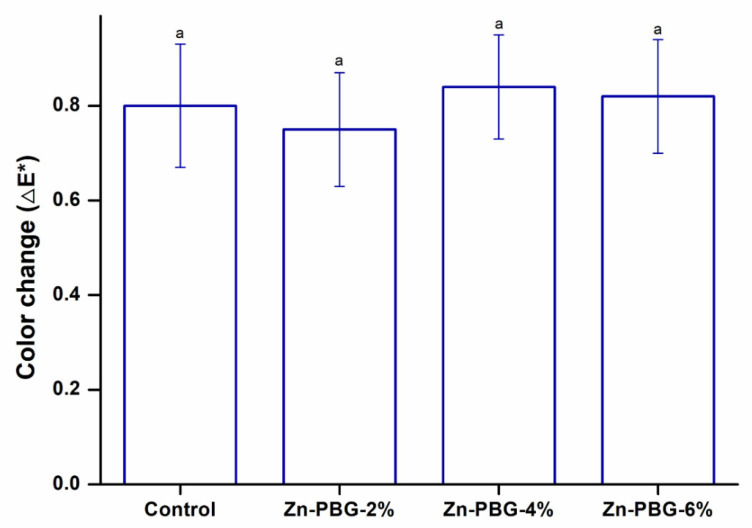
Color change of the control, Zn-PBG-2%, Zn-PBG-4%, and Zn-PBG-6% groups. The lowercase letter indicates no significant difference between the control and experimental groups (*p* > 0.05).

Denture color and gloss are also clinically important factors [32]. Color changes can be assessed using a visual method or a colorimeter [33]. Observing discoloration with the naked eye has the disadvantage that the subjectivity of the inspector cannot be excluded, so using a colorimeter is the preferred method [34]. The means and standard deviations of the color differences for each group are shown in Figure 3. The results reveal that there were no significant differences between the control and experimental groups. According to the numerical values quantified by the National Bureau of Standards (Washington, DC, USA), there is no clinical problem if the ΔE* value is no greater than 0.92. The color change experiment in this study resulted in ΔE* values of 0.80, 0.77, 0.84, and 0.82 for the 2 wt%, 4 wt%, 6 wt%, and control groups, respectively, indicating that the color change value did not affect the clinical acceptability [33,35].

**Figure 4:**
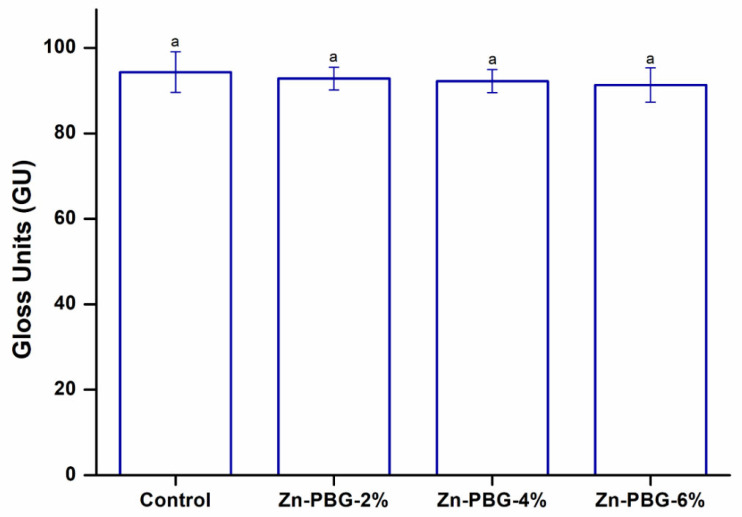
Gloss units of the control, Zn-PBG-2%, Zn-PBG-4%, and Zn-PBG-6% groups. The lowercase letter indicates no significant difference between the control and experimental groups (*p* > 0.05).

The gloss of a material surface is an optical phenomenon expressed as the amount of light reflected from the surface [36]. Therefore, the reflectance varies according to the angle of incidence, and the glossiness that affects aesthetics also differs depending on the surface properties. Therefore, it is important to measure gloss when assessing surface properties [37]. The gloss values for all the groups were more than 90 gloss units (Figure 4). In addition, the control group showed no significant difference from the experimental groups (*p* > 0.05).

**Figure 5:**
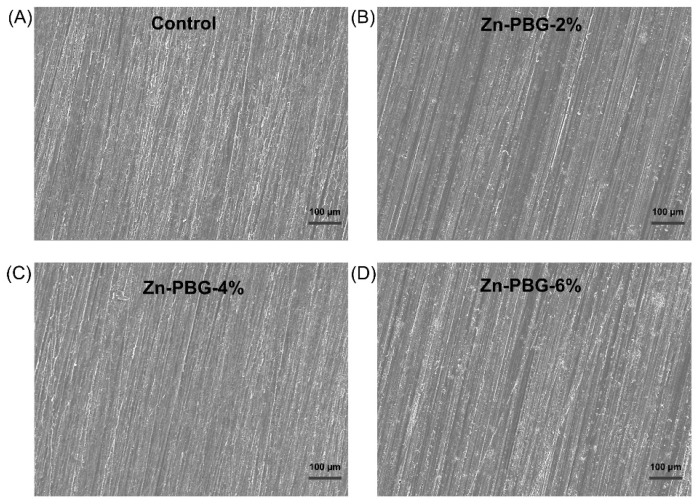
Representative SEM micrographs of the sample surfaces; (**A**) Control, (**B**) Zn-PBG-2%, (**C**) Zn-PBG-4%, (**D**) Zn-PBG-6%.

As shown in Figure 5, the SEM images of the control and experimental groups indicate that no significant differences were present in the surface morphology.

The first null hypothesis, that a denture-relining material containing zinc-doped phosphate glass would not result in significant differences in the surface properties compared to the control, was accepted.

Toxicity due to denture base materials may cause irritation, inflammation, and allergic reactions in oral tissues [1]. It is also reported that toxicity can arise from the local effects of toxic substances released from the denture base [38]. To prevent these side effects, it is important to determine whether toxic substances are released from denture base materials as a result of contact with oral mucosa or saliva [21]. Materials should therefore exhibit excellent biocompatibility for long-term use in clinical practice, and the MTT assay was performed to test this [1]. This method measures the activity of cellular dehydrogenase, which converts the tetrazolium salt into an insoluble formazan compound [39]. The metabolic activity of cells can be determined by measuring the amount of formazan produced through cell experiments. [1,39]. Therefore, it is a useful method for evaluating the toxicity of dental materials. Consequently, cytotoxicity was not observed for all groups.

**Figure 6:**
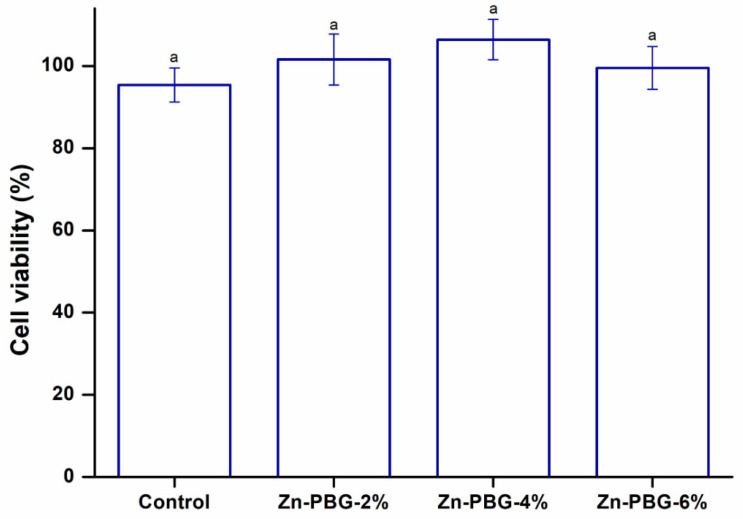
Cell viability of the control, Zn-PBG-2%, Zn-PBG-4%, and Zn-PBG-6% groups. The lowercase letter indicates that there is no significant difference between the control and experimental groups (*p* > 0.05).

As shown by the MTT assay results in Figure 6, there was no statistically significant difference in the cell viability between the control and experimental groups.

The second null hypothesis, that a denture-relining material containing zinc-doped phosphate glass would not result in significant differences in cell cytotoxicity compared to the control, was accepted.

**Figure 7:**
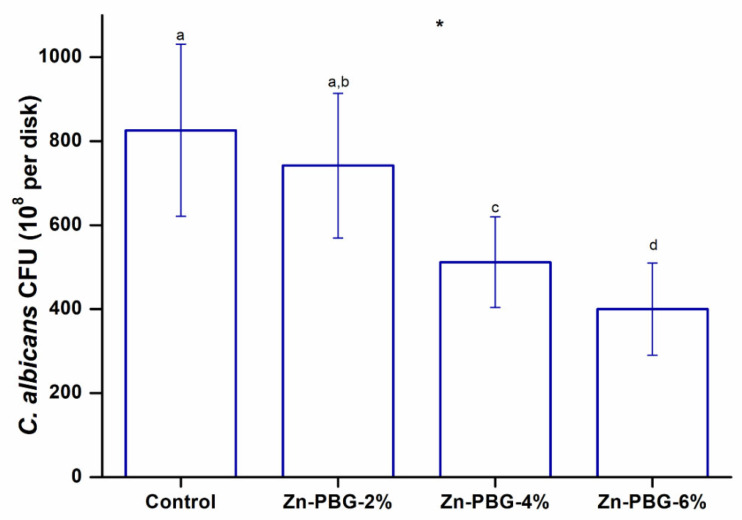
*C. albicans* CFU counts of the control, Zn-PBG-2%, Zn-PBG-4%, and Zn-PBG-6% groups. The lowercase letters indicate that there were significant differences between the control and experimental groups. * *p* < 0.05 for the comparison between all groups.

Fungi readily attach to denture surfaces, and the resulting accumulation of fungi is a major problem for denture wearers [18]. Fungi colonized on the surface of dentures cause denture stomatitis, and it has been reported that this is particularly problematic for the elderly and patients with reduced salivation [7,20]. Several types of microorganisms can colonize the denture surface, but *Candida albicans*, known to be the pathogen most related to denture stomatitis, was targeted [27]. The adhesion of *C. albicans* to the surface is an important step in developing denture stomatitis [1,6,18]. The results of evaluating the antifungal efficacy of the test materials are shown in Figure 7. The CFU counts for the 2, 4, and 6 wt% Zn-doped glass samples were significantly lower than those of the control group. The 6 wt% samples showed the lowest average fungal count, which was half that of the control group. Zn-PBG is therefore expected to exhibit strong antifungal activity [23]. These findings indicate the effect of cations on raising the ambient pH and the intracellular invasion of zinc ions that cause cell membrane destruction [25]. Overall, the results demonstrate that the ability of the denture-relining material coated with zinc-doped phosphate glass to inhibit fungal adhesion was significantly greater than that of the control.

The third null hypothesis, that a denture-relining material containing zinc-doped phosphate glass would not result in significant differences in the antifungal activity compared to the control, was rejected.

In this study, phosphate-based glass containing zinc was investigated. Zinc possesses antifungal properties, although its mode of action is currently unclear. Previous studies have shown that zinc can induce oxidative stress and cause protein dysfunction in microorganisms. In an earlier study, zinc significantly decreased the number of Gram-negative and Gram-positive bacteria [22,24]. Its encapsulation in phosphate-based glass allows for the control of degradation and the ion release rate, which are key properties of this new material, in order to maintain antifungal activity [25].

Only one type of denture base resin and *Candida albicans* were tested in this study; therefore, there is a limitation in the design of various variables in the experiment. More meaningful results can be obtained if future investigations are conducted using different types of denture base resins and multiple clinically relevant microorganisms. Moreover, a follow-up study to observe the antifungal effect over time is necessary, including the elucidation of a clear antifungal action mechanism for clinical application. The denture base resin developed in this study is bio-friendly and exhibits antifungal activity without changing the properties of the material surface, making it a promising material for denture users in dental clinics.

## 4. Conclusions

Zn-PBG is a promising antifungal nanomaterial for use as a size-adjustable filler for denture base resins. A denture-relining material coated with zinc-doped phosphate glass showed antifungal efficacy toward *Candida albicans*. In addition, it was shown that surface properties, such as contact angle, microhardness, color difference, and gloss, were not significantly different compared to the control group. In the biocompatibility tests, the experimental groups did not exhibit cytotoxicity. The present study investigated the antifungal efficacy of a denture-relining material coated with zinc-doped phosphate glass over a short period. Therefore, a long-term investigation is needed. A novel denture-relining material prepared with zinc-doped phosphate glass has potential as an antifungal dental material that is biocompatible while retaining its surface properties without deteriorating.

## Data Availability

Not applicable.
